# Comparison of short-channel separation and spatial domain filtering for removal of non-neural components in functional near-infrared spectroscopy signals

**DOI:** 10.1117/1.NPh.8.1.015004

**Published:** 2021-02-13

**Authors:** J. Adam Noah, Xian Zhang, Swethasri Dravida, Courtney DiCocco, Tatsuya Suzuki, Richard N. Aslin, Ilias Tachtsidis, Joy Hirsch

**Affiliations:** a Yale School of Medicine, Department of Psychiatry, Brain Function Laboratory, New Haven, Connecticut, United States; b Yale School of Medicine, Interdepartmental Neuroscience Program New Haven, Connecticut, United States; c Yale School of Medicine, Brain Function Laboratory, New Haven, Connecticut, United States; d Meiji University, Graduate School of Science and Technology, Electrical Engineering Program, Kawasaki, Japan; e Meiji University, School of Science and Technology, Department of Electronics and Bioinformatics, Kawasaki, Japan; f Haskins Laboratories, New Haven, Connecticut, United States; g Yale University, Department of Psychology, New Haven, Connecticut, United States; h University College London, Department of Medical Physics and Biomedical Engineering, London, United Kingdom; i Yale School of Medicine, Department of Neuroscience, New Haven, Connecticut, United States; j Yale School of Medicine, Department of Comparative Medicine, New Haven, Connecticut, United States

**Keywords:** functional near-infrared spectroscopy, systemic artifact, spatial filter, short channel

## Abstract

**Significance:** With the increasing popularity of functional near-infrared spectroscopy (fNIRS), the need to determine localization of the source and nature of the signals has grown.

**Aim:** We compare strategies for removal of non-neural signals for a finger-thumb tapping task, which shows responses in contralateral motor cortex and a visual checkerboard viewing task that produces activity within the occipital lobe.

**Approach:** We compare temporal regression strategies using short-channel separation to a spatial principal component (PC) filter that removes global signals present in all channels. For short-channel temporal regression, we compare non-neural signal removal using first and combined first and second PCs from a broad distribution of short channels to limited distribution on the forehead.

**Results:** Temporal regression of non-neural information from broadly distributed short channels did not differ from forehead-only distribution. Spatial PC filtering provides results similar to short-channel separation using the temporal domain. Utilizing both first and second PCs from short channels removes additional non-neural information.

**Conclusions:** We conclude that short-channel information in the temporal domain and spatial domain regression filtering methods remove similar non-neural components represented in scalp hemodynamics from fNIRS signals and that either technique is sufficient to remove non-neural components.

## 1 Introduction

Functional near-infrared spectroscopy (fNIRS) has emerged as a widely applied neuroimaging technique for cognitive and two-person social neuroscience since its early adoption as a brain monitoring tool for infants.^1^^–^^4^ In recent years, multi-channel instruments have been developed that allow for functional recordings of the entire superficial cortex.^5^^–^^8^ While fNIRS has limited spatial resolution and sensitivity to nonsuperficial structures of the cortex compared to functional magnetic resonance imaging (fMRI), it provides a neural imaging modality that allows measurements from subjects that are performing complex, ecologically valid tasks including locomotion, dancing, talking, and social interaction.^7^^,^^9^^–^^14^ Additionally, fNIRS can be used for functional imaging of individuals who may be contraindicated for scanning in the high magnetic field of fMRI.^15^^–^^20^

Even with this growth in usage and functionality, there are caveats that need to be taken into consideration. fNIRS is a brain imaging tool that quantifies relative changes in the spectral absorption of hemoglobin chromophores related to hemodynamic/oxygenation activity. This activity is a proxy for neural function, similar to the blood oxygen level dependent (BOLD) signal in fMRI. The spatial resolution of conventional multi-channel fNIRS devices with 3 cm (long) source–detector distances is roughly 3 cm with a depth of penetration of only the superficial cortex (reported to correlate best with BOLD at 14 mm from the scalp).^21^ This resolution is significantly reduced from that of fMRI. Additionally, fNIRS signals are recorded at the surface of the head hence they interrogate a mix volume of tissue that includes both the scalp, skull, cerebrospinal fluid, and the brain. This means that the relative changes in spectral absorption of the hemoglobin differences are not specific to only the cortex, but recorded signals also contain components related to hemodynamics in the scalp.^5^^,^^22^^–^^25^ The hemodynamic fluctuations in the scalp are particularly problematic as fNIRS is targeted at real-world motor and social tasks, such as speech,^26^^–^^28^ that cannot be performed in fMRI. Real-world tasks also depend on the cognitive and emotional state of the individual, which has been shown to influence oxyhemoglobin (OxyHb) signals in the scalp. These changes in oxygenation in the scalp are particularly susceptible to task-evoked sympathetic arterial vasoconstriction followed by a decrease in venous volume, which contribute to non-neural hemodynamics^23^ recorded by long fNIRS channels. Removal or regression of the scalp signal, which has been argued to comprise the largest non-neural signal component in long fNIRS channels,^22^^,^^29^ has posed a challenge to the fNIRS community. Methods utilizing several temporal and spatial regression strategies have been proposed to address this contaminating effect.

Systemic physiological changes, such as respiration rate, blood pressure, partial pressure of ${\mathrm{CO}}_{2}$, and heart rate, can also influence the hemodynamic/oxygenation changes of both the scalp and the brain.^22^^,^^23^^,^^29^ Early attempts to separate signal components associated with changes in non-neural hemodynamics from neural/cortical signals rely largely on removing temporal components embedded in the signal.^29^ Removal strategies include principal component (PC) and wavelet regression to remove the first two PCs during a resting-state task.^30^^–^^32^ Others have regressed temporal information from laser Doppler and other physiological recordings to determine specific components of the signal related to scalp blood flow.^27^^,^^32^^–^^37^ Additional methods utilizing temporal regression have been developed to separate the systemic responses from cortical activity during the task. Another recently developed method to separate non-neural information in the temporal domain is short-channel separation.^38^^–^^48^ The goal of short-channel separation is to obtain specific scalp-only hemodynamic recordings and to subsequently remove this non-neural information from long-channel recordings. Numerous studies have utilized short-channel regression to separate the hemodynamic responses from these surface non-neural components from the deeper cortical sources and have reported significant improvements in the spatial localization of OxyHb signals, but less so for deoxyhemoglobin (DeOxyHb).^27^^,^^42^^,^^44^ Sato et al.^44^ specifically showed that removal of the first PC of four short-channel recordings can be utilized to significantly improve spatial specificity of tasks that involve digit manipulation. This study did not attempt to specify the optimal scalp locations of short channels, but rather showed that utilization of the linear regression of the first PC of short-channel recordings within the region was sufficient to remove systemic components from the OxyHb signal. A follow-up study showed that the first two PCs of four short-channel recordings could theoretically be utilized to regress motion artifact in addition to systemic components.^42^ While the PC regression method proposed by Sato et al. was able to increase the spatial specificity of motor tasks in patients and healthy participants, there is still no consensus as to the gold standard method to remove scalp and non-neural hemodynamics through the utilization of short-channel recordings.

Others have argued that it is necessary to record short-channel information as well as additional sources of physiology from multiple regions of the scalp to determine local influences of scalp hemodynamics and additionally remove them.^24^^,^^49^^,^^50^ Another recent method for removing systemic information has been proposed that utilizes multi-distance tomographic recordings to separate superficial from cortical hemodynamics.^41^ The effectiveness of short-channel regression has been further investigated and compared to recently developed methods that are also intended to separate systemic from cortical responses in fNIRS recordings.^51^^,^^52^ Studies utilizing multimodal recordings of systemic responses have been used to create improved methods of physiological noise regression from measures independent of the optical fNIRS signals by expanding on early studies that used single systemic recordings to regress non-neural components.^33^ von Lühmann et al.^52^ developed a method that utilized multiple systemic recordings, including blood pressure, blood volume (photoplethysmography), respiration, and movement (via accelerometer), to regress systemic components from fNIRS signals using temporal information from the signals. They concluded that their approach was computationally efficient and improved the robustness of hemodynamic response estimation and could also be used to improve brain–machine interfaces that utilize fNIRS.^52^

In contrast to temporal domain regression methods, a separate approach utilizes spatial domain information across multiple channels to determine a spatial similarity matrix that can be utilized to regress homologous spatial information that is assumed to be non-neural in origin from cortical signals.^32^ An early attempt to determine a spatial correlation matrix to regress blood pressure and respiration signals using an eigenvector regression approach was developed by Zhang et al.^32^ This approach collected a separate short baseline or resting-state set of fNIRS data independent from the task collection. A spatial correlation matrix was determined and assumed to be representative of the shared spatial pattern of systemic hemodynamics in the fNIRS recordings. The first two eigenvectors of the recordings were determined and regressed from the long channels based on the spatial matrix. This approach was shown to have potential in increasing spatial sensitivity to motor tasks, but also revealed some additional questions. While the study by Zhang et al. had potential to remove global signal and increase spatial sensitivity, it also appears to introduce some potential for a negative artifact in the contralateral superficial cortex. The 2016 study by Sato et al.^44^ further confirmed this contralateral artifact. Sato et al. tested the approach developed by Zhang et al.^32^ (referred to as RestEV) and compared it to short-channel regression and gold standard fMRI approaches. The same contralateral negative artifact was present but not seen in short-channel regression or fMRI. We have developed an additional approach that uses spatial Gaussian filtering on long-channel recordings.^28^^,^^53^ This method has been validated for improving the spatial sensitivity of fNIRS recordings in motor, visual, and speaking tasks and has also been utilized for two-person interactions including drumming, eye contact, and decision making.^7^^,^^10^^,^^11^^,^^54^^–^^56^ Specific benefits of this spatial filtering method are that it is performed on the entire task-based signals and not on short baseline or separate resting-state data. In addition, the spatial Gaussian filtering is performed using positional information from the three-dimensional (3D) coordinates of digitized channels. This technique allows us to determine the specific systemic responses to individual tasks including speech, social, and motor interactions, which are commonly studied using fNIRS. This is important as the number of research studies utilizing fNIRS in speaking and motor behavior tasks is increasing dramatically each year.^57^

While active speech and motor tasks are common paradigms targeted at skin and systemic component removal methods, other passive tasks involving vision and listening to speech may also have increased skin response in long fNIRS channels.^58^ How the state of the individual differentially affects hemoglobin signals in the skin during active and passive tasks is also unknown. While many active tasks including motor behaviors and verbal fluency can generate large skin hemodynamics in some individuals, it has been reported that little systemic influence is present in recordings from adults and infants participating in tasks that involve passive viewing stimuli of faces.^38^^,^^58^ In these experiments, the removal of skin hemodynamics did not change the statistical inferences drawn from the recorded long-channel data. This suggests that sources of systemic, non-neural information including blood pressure and blood flow associated with active speech and digit manipulation tasks^27^^,^^28^^,^^32^^,^^44^^,^^50^^–^^53^ may not have the same effect on skin hemodynamics as passive viewing of visual or auditory stimuli.

Our goal in this study is to compare fNIRS signals recorded during two fiducial tasks, one passive and one active, that are known to elicit robust and spatially specific responses. We will compare signals before and after short-channel regression in the temporal domain versus the spatial PC filter developed by Zhang et al.^53^ We will specifically compare how the first and second PC of combined short channels can be utilized for non-neural component regression as well as the similarity between the regressed signals. We will also compare regression methods using a broad spatial distribution of short channels arranged throughout the entire scalp to include localized hemodynamic responses to a restricted distribution of short channels placed only on the forehead of subjects. We seek to determine whether task-based spatial PC filtering will extract non-neural components that are not significantly different from those extracted from short-channel regression regardless of the placement of the short-channel optodes.

## 2 Methods

### 2.1 Participants

Seven healthy adults (57.1% female; median age 31 years (range 24 to 71); 100% right-handed^59^) participated in the study consisting of two fNIRS tasks: finger-thumb tapping and passive viewing of a reversing checkerboard. Short-channel data were collected in both tasks. All participants provided written informed consent in accordance with guidelines approved by the Yale University Human Investigation Committee (HIC #1501015178).

### 2.2 Paradigm

Each subject participated in two separate data collection sessions. Each session was identical except for the positioning of the short-channel optodes. During the first data collection session, each participant took part in two tasks. The first was a passive visual task in which the subject was shown a reversing checkerboard stimulus presented using a custom python script in PsychoPy 2.7.^60^ The checkerboard stimulus alternated between black and white configurations at 7 Hz and was presented on a 27 in., 16×9 monitor placed approximately 70 cm away from the participant (subtending 12.3 deg of visual angle). The stimulus was presented in a block design in which the phase-alternating checkerboard was viewed for 15 s followed by a stationary blank screen with a fixation cross at the center for 15 s. Each participant watched 4 alternating checkerboard and blank blocks for a total of 2 min, followed by a second repetition of the entire procedure.

The second task was right-handed finger-thumb tapping. Participants were shown in random order the numbers 1 to 4 on the screen. If the number was 1, subjects were asked to firmly tap their pointer finger to their thumb. If the participants saw number 2, they were asked to tap their middle finger to thumb, 3 was ring finger to thumb, and 4 was pinky finger to thumb. The task was presented in a block design with numbers appearing one per second for 15 s total. The active portion of the task alternated with a 15-s rest period in which subjects were asked to focus on a fixation cross in the center of the screen. The alternating blocks of finger-thumb tapping and rest were repeated 6 times for a total of 3 min. The task was then repeated for a total session of 6 min.

**Fig. 1:**
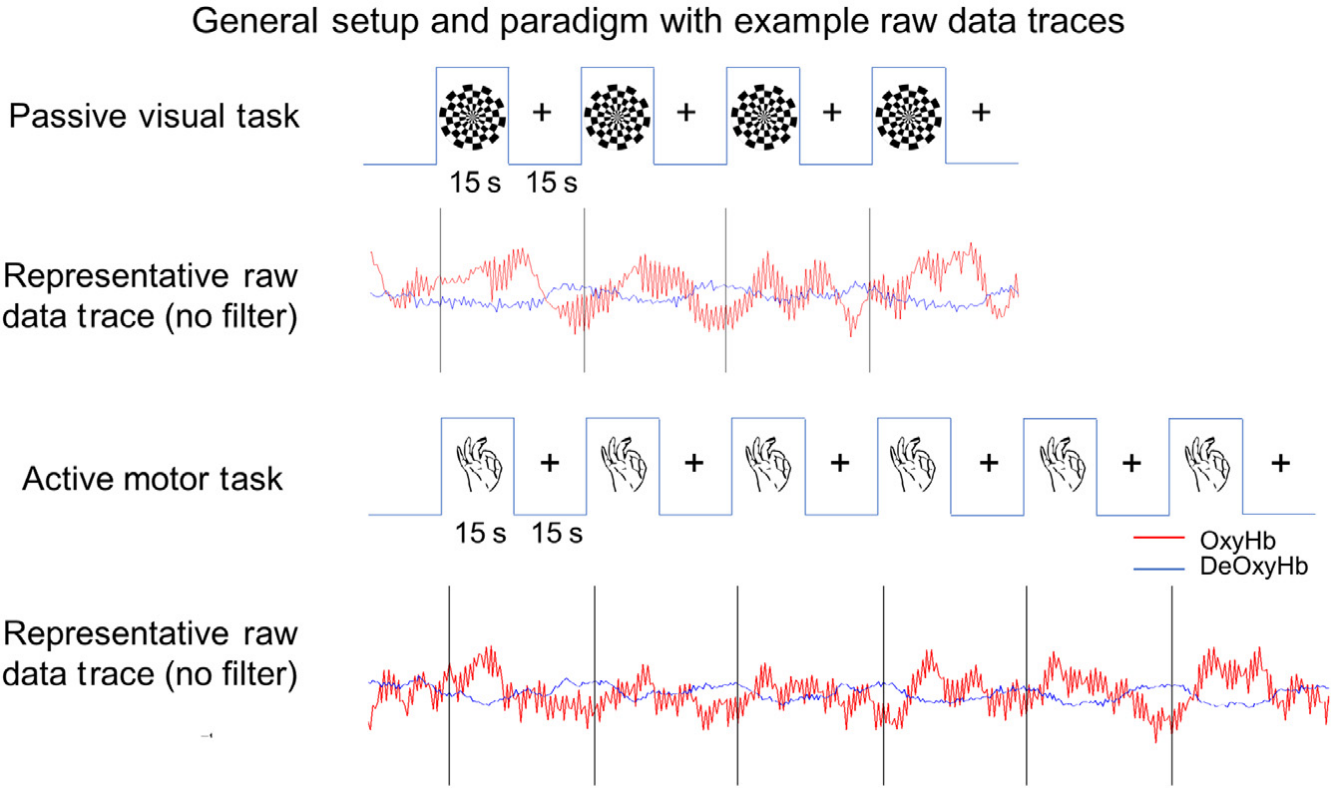
Task paradigm and example raw data. The upper diagram represents the passive visual stimulus. Subjects watched a reversing checkerboard pattern for a total of 2 min. The task shown was repeated twice. The example raw data trace below shows both OxyHb (red) and DeOxyHb (blue) signals in a channel in the expected ROI prior to any signal processing. The finger-thumb tapping motor task is shown below. The task was a total of 3 min and was repeated twice. The example OxyHb (red) and DeOxyHb (blue) signals from a channel in the expected contralateral motor cortex are shown below.

A diagrammatic representation of both tasks as well as sample raw data traces from channels within the expected region of interest (ROI) (occipital lobe for visual task and left motor cortex for finger tapping) is shown in Fig. 1.

### 2.3 Equipment

**Fig. 2:**
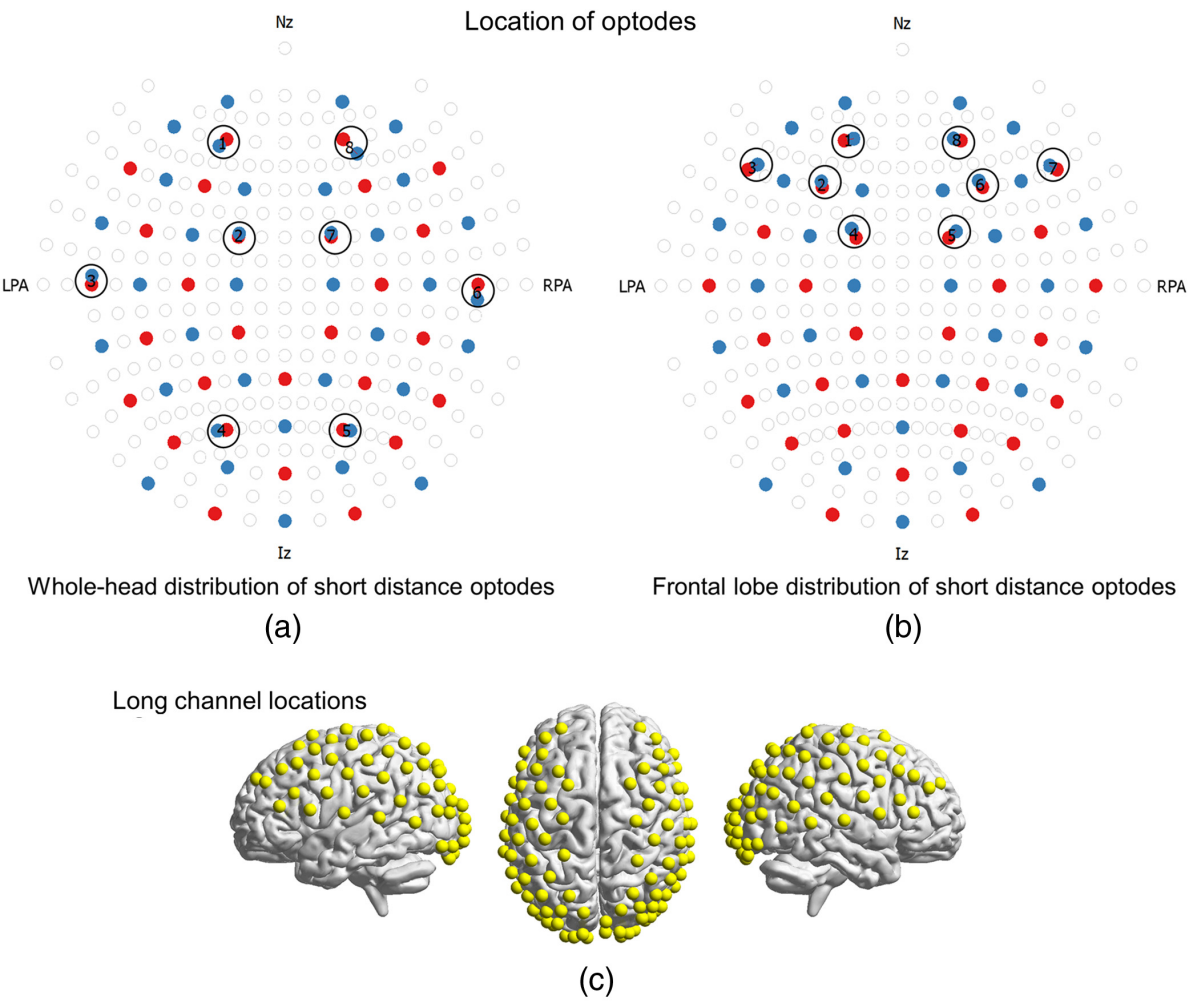
Placement of optodes and locations for short and long channels. Location of emitter (red) and detector (blue) optodes with respect to 10-20 is shown in (a) for the broad distribution of short channels (locations outlined with black circles) and in (b) for the frontal lobe only distribution (locations outlined with black circles). Short optodes were a special hardware fiber provided by NIRx that coupled 8-mm separated fibers to standard emitter optodes. The placement of the long (3 cm) optode pairs was the same for both experiments. (c) The location of the median location for long channels from the seven subjects is rendered on a standard brain template. Individual channel locations are shown as yellow spheres on the cortex.

A NIRx NIRScout system was used to collect raw optical density variations in the 760- and 850-nm signals at a frequency of 2.08 Hz. Data were obtained using a custom cap in which 31 emitter optodes were arranged with 30 detector optodes as well as one short-channel coupler providing 8 individual short-channel recordings. Standard 3-cm optode distances were used for long channels. The placement of optodes within the 10-20 system is shown in Figs. 2(a) and 2(b). The location of the long channels is shown in the rendering in Fig. 2(c). Short channels had 8 mm of separation between emitter and detector optodes. The NIRx NIRScout uses a time pulsing system that allows for a specialized optode coupler to be attached to a single detector optode. The short-channel coupler consisted of eight individual fiber bundles. The positions of each short optode detector are shown inside the black circles in Figs. 2(a) and 2(b).

As stated above, two separate short-channel procedures were performed on each subject on separate days. The first included placement of short channels broadly distributed across the head to cover the entire superficial cortex [Fig. 2(a)]. The second arrangement placed all the short channels on the forehead only [Fig. 2(b)]. These two arrangements of short channels were designed to address the secondary goal of the study: to determine whether the spatial distribution and number of short channels provided an optimal correction for the confounding effects of localized surface vascular signals. This is an important question as it is faster and more convenient to place short channels on the forehead rather than on areas of the scalp covered by hair, thereby eliminating the longer setup times associated with making good optode contact.

Prior to each recording session, each optode was placed independently using methods described previously.^7^^,^^14^ Briefly, a lighted fiber optic probe (Daiso, Japan) was used to move all hair from each optode holder. The spring loaded optode and cap were placed in the holder and upon placing all optodes a calibration procedure was performed to assure each channel was recording expected quantities of light. To assure consistency, placement of the most anterior optode holder on the cap (left blank in this study) was placed 1 cm above nasion. Anatomical locations of optodes in relation to standard head landmarks were determined for each participant using a Patriot 3D Digitizer (Polhemus, Colchester, Vermont).^61^^–^^65^ Montreal Neurological Institute (MNI) coordinates^66^ for each channel were obtained using NIRS-SPM software,^67^ and the corresponding anatomical locations of each channel are shown in Fig. 2. These locations are detailed in Table S1 in the Supplemental Material, which lists the group median MNI coordinates and anatomical regions with probability estimates for each of the channels.

**Table 1:** Similarity in results comparing spatial to temporal filtering methods when short channels are widely distributed. For all subjects, we compared the similarity of the short-channel regressions (PC1 versus PC1 + PC2) to the spatial PC method when short channels were placed broadly on the scalp. The similarity index ranges from 0 to 1 in which a value of 1 represented identical signals and 0 represented no similarity between signals. We compared the channel showing the largest t-value to the GLM analysis within the motor and visual ROI for similarity in signal after performing either spatial or temporal regression. OxyHb (top) and DeOxyHb comparisons are made for both visual stimuli (left) and motor tasks (right). Short-channel PC 1 regression is compared to spatial PC regression for DeOxyHb and for OxyHb. Subject 2 shows a dramatic increase in similarity between temporal and spatial regression when including PC2 as well as PC1 for short-channel separation.

Whole head									
Passive-viewing task					Finger-thumb tapping task				
Subject	PC1	PC1	PC1 and PC2	PC1 and PC2	Subject	PC1	PC1	PC1 and PC2	PC1 and PC2
	Oxy	DeOxy	Oxy	DeOxy		Oxy	DeOxy	Oxy	DeOxy
1	0.963	0.996	0.974	0.996	1	0.940	0.950	0.949	0.947
2	0.966	0.984	0.976	0.982	2	0.690	0.960	0.869	0.961
3	0.907	0.959	0.948	0.963	3	0.960	0.980	0.976	0.984
4	0.950	0.929	0.953	0.963	4	0.970	0.970	0.979	0.973
5	0.987	0.951	0.988	0.951	5	0.960	0.990	0.951	0.984
6	0.905	0.972	0.944	0.972	6	0.970	0.980	0.984	0.984
7	0.791	0.979	0.792	0.977	7	0.970	0.990	0.971	0.991

### 2.4 Signal Processing

#### 2.4.1 Preprocessing

Raw optical density variations recorded from the NIRx NIRScout were converted into changes in relative chromophore concentrations using the Beer–Lambert law.^68^^–^^70^ Baseline drift was removed using wavelet detrending provided in NIRS-SPM.^67^ Little to no motion artifact was present in the data and thus, no motion rejection filtering was performed on the data. Additionally, no bandpass filter was used prior to subsequent processing. Example raw data traces (prior to systemic filtering or block averaging of task) are shown in Fig. 1 for reference.

Data were further processed using one of two general linear model (GLM) methods to regress the superficial component separately from the task-induced signal. The first method utilized short-channel information to determine the systemic regressor, and the second method utilized a spatial PC filter.^28^^,^^53^ All GLM regression analyses were performed on the entire trace of both Oxy- and DeOxyHb signals.

#### 2.4.2 Short-channel data processing

The first two PCs of the combined signals of the eight short channels were used for GLM regression from the Oxy- and DeOxyHb signals for each subject. In some instances, because of hair and other scalp contact issues, all eight short channels were not usable. A minimum of four short channels were required to obtain two PCs for subsequent analyses regardless of broad or frontal placement of short optodes.

The raw signals, the superficial component, and the filtered data were determined for each subject. For the PC analysis on short channels, a matrix of 8 channels by N sample points was determined. The PC analyses only use the first one or two major PCs for short-channel regression. In the case of using two PCs, X represents a three-column matrix, where the first two columns are the waveform of the two PCs derived from the short channels. The last column is a constant 1 for the intercept term. Y represents the raw data of a standard channel. Linear regression was performed using the regress function in MATLAB^®^: [Beta, ∼, Residual] = regress(Y,X), where Beta is the coefficient of short-channel PC in the raw data and the Residual is the cleaned or filtered data or the fNIRS data with short-channel PCs being regressed.

#### 2.4.3 Task-based spatial regression

The task-based spatial PC regression strategy has been published previously.^28^^,^^53^ Here, we provide the essence of the spatial filter method for removing superficial skin influences from long fNIRS channels as the following.

Each channel is associated with a sphere coordinate (θ,ψ,R) converted from MNI coordinates obtained from 3D digitizing and centered on the anterior commissure. For simplicity, the variation in R is ignored. The global component is the result of a Gaussian spatial smoothing on a two-dimensional sphere. The radius of Gaussian smoothing kernel set to 0.8 rad or 46 deg.^28^^,^^53^ The filtered data are the difference between the raw data and the global or superficial component.

For each channel i, the global or superficial component

$${\text{Global component}}_{i}=\overset{\text{number of channel}}{\underset{j=1}{\sum }}w_{ij}{\text{Data}}_{j}.$$

The larger the distance between channel i and channel j, smaller the $w_{ij}$ value according to the two-dimensional Gaussian function.

$w_{ij}=e^{\frac{-{\text{distance}}_{ij}^{2}}{2r^{2}}}$ r=0.8; the sum of $w_{ij}$ is normalized to 1.

Once the superficial component is determined, it is subtracted from the raw data to yield the cortical data.

#### 2.4.4 Hemodynamic modeling

Hemodynamic responses for filtered or cleaned OxyHb and DeOxyHb signals were compared between task and rest conditions using a GLM procedure in NIRS-SPM.^67^ Event epochs within the time series were convolved with a standard hemodynamic response function and were fit to the data, providing individual “beta values” for both chromophores for each channel across all conditions.

### 2.5 Signal Comparison in Regions of Interest

To compare the effect of the spatial and temporal GLM filtering techniques, we first determined the channel with the most significant fit to the convolved hemodynamic response to the task (Sec. 2.4.4) within the expected ROI. This was performed for spatial and all short-channel regression methods. For the finger-thumb tapping task, this ROI was the contralateral (left) motor cortex and for the visual task, this was the bilateral occipital lobe. Channel locations were determined based on 3D coordinates obtained with the Polhemus Patriot Digitizer in the AAL atlas.^71^ To assure we were not overly conservative in the ROI we used, a broad definition, which included any channel having 20% or more probability of being located in the specified ROI for the task, was considered for comparison. The amplitude of Oxy- and DeOxyHb signals for each subject were determined using GLM for each responding channel. For any specific subject, a t-value threshold of 5 or greater for a channel was accepted (p<0.00001) as significant activity compared to rest. No maximum significant channel per subject differed across spatial or temporal techniques or between OxyHb and DeOxyHb results.

#### 2.5.1 Correlation between adjusted data from spatial and temporal methods

We compared filtering techniques through a similarity index in which the spatial PC filter was compared to the temporal filter using short-channel regression of the first and combined first and second PCs of the combined short channels from widely distributed and from frontal lobe only short channels. The similarity between the spatial and temporal regression methods was determined in MATLAB R2019b using the correlation function (corrcoef). For both visual and motor tasks on each subject, we compared the entire adjusted (i.e., temporal or spatial filtered) signal for both Oxy- and DeOxyHb. The similarity index represented how similar the two (temporal or spatial) filtering methods were to each other on a scale of 0 to 1. In the case of short-channel adjusted signals, we determined the similarity for both first PC as well as combined first and second PC regression strategies to the adjusted signals determined from the spatial filter. This same comparison with the spatial regression method was performed for both broad and frontal only short-channel optode arrays.

## 3 Results

### 3.1 Comparison of Spatial and Temporal Methods using Wide Distribution of Short-Channel Optodes

#### 3.1.1 Visual task

**Fig. 3:**
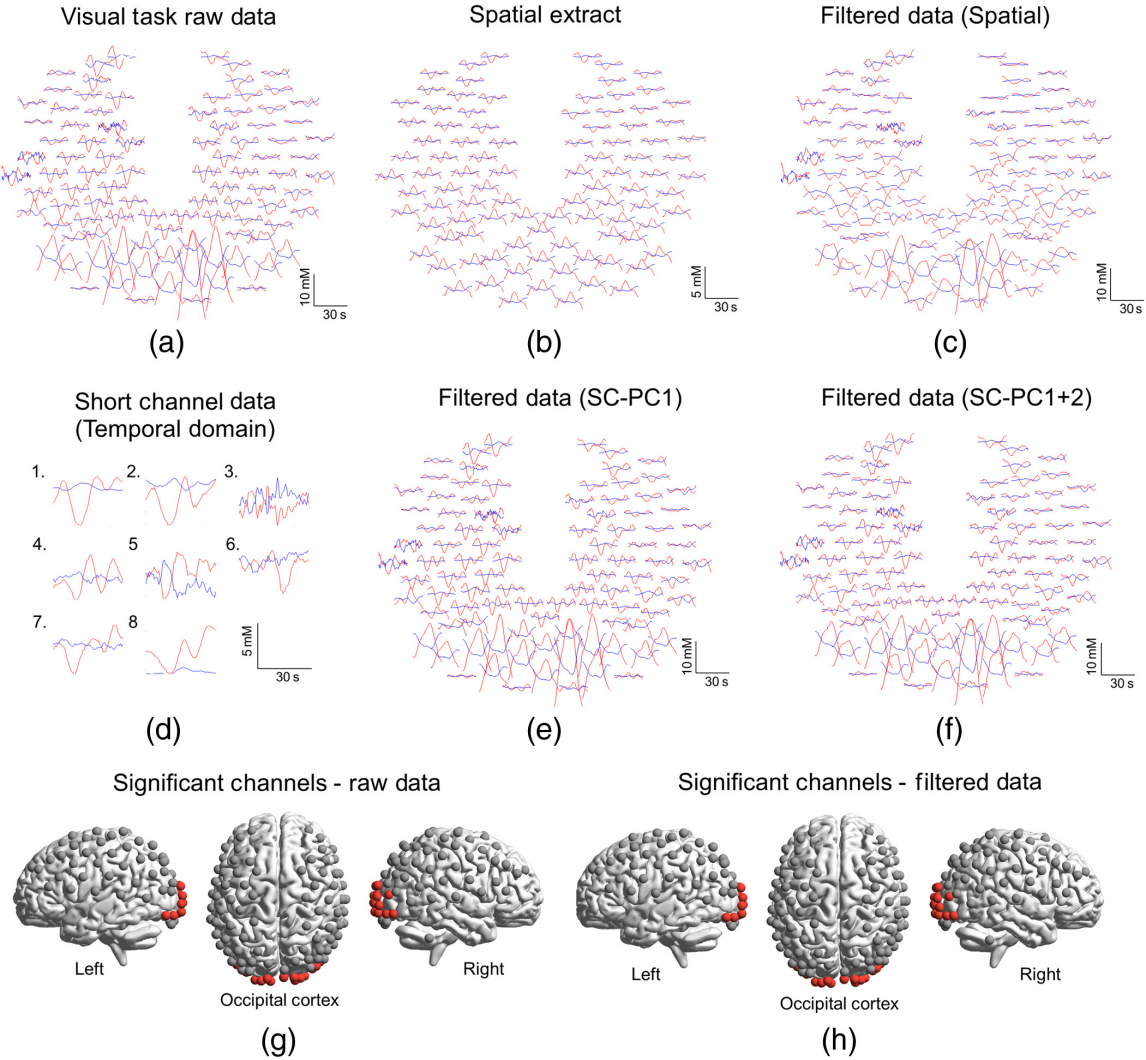
Selected visual responses from representative participant comparing event-triggered average data between short-channel temporal filtering to spatial PC filter. (a) Raw Oxy- and DeOxyHb (red and blue, respectively) signals event-triggered and averaged across the four blocks of the flashing checkerboard stimulus. The layout of all plots shows the channels in the two-dimensional 10-20 arrangement from Fig. 2. The top of the figure represents the front of the head and the bottom of the figure represents the back of the head or the occipital lobe. The expected Oxy- and DeOxyHb hemodynamic separation is seen localized in the occipital lobe. Additional noise is present in other channels throughout the cortex. (b) The spatial PC filter extracted component. The spatial PC filter determines a global or nonspatially specific component present in the fNIRS signals and removes it based on spatial domain information only. (c) The resultant of removing the non-neural extract shown in (b) from the raw data in (a). The globally uniform nature of the signal is reduced and expected hemodynamic responses are localized to the occipital lobe. (d) Event-triggered average responses from the eight individual short channels. (e) Resultant of regressing the first PC of the combined short channels shown in (d) from the raw data in (a). (f) Resultant of regressing the first and second PCs of the combined short channels shown in (d) from (a). Channels showing significant response for (g) raw and (h) filtered data. No differences in the number or spatial location of significant channels were found using either spatial or temporal filtering. The results in (h), therefore, represent the results of both filtering techniques as they are equivalent. To determine significance of response single, subject data were modeled using a hemodynamic response function provided in SPM8. In the case that OxyHb and DeOxyHb signals, both reached a threshold of a t-value of 5 or greater channels are marked as red. For visual stimuli, significant channel results were identical for filtered and raw data. The data from subject 3 shown in Fig. 3 represent exemplar but consistent results from all subjects that participated in the study for the passive visual task.

The hemodynamic results from the passive visual stimulus are shown for a single representative subject in Fig. 3. This subject displayed hemodynamic signals that were similar to all the other subjects in the experiment for the visual task. Figure 3(a) is a plot of the raw Oxy- and DeOxyHb signals shown in the same configuration as the 10-20 layout seen in Fig. 2(a). Red traces represent OxyHb signals and blue traces represent DeOxyHb signals. The top of the figure is the anterior and the bottom is the posterior of the head. The large hemodynamic response to the visual stimulus is clearly visible in the posterior traces located over the occipital lobe. A superficial (i.e., non-neural) and more diffusely localized response is seen most prominently in the middle channels located between frontal and occipital lobes. Figure 3(b) shows the superficial signal extracted using the spatial PC filter. The global nature of the signal is pronounced, and as expected, no spatially specific response can be seen in the extracted signal. Figure 3(c) shows the resulting adjusted Oxy- and DeOxyHb traces after separating the spatially filtered responses shown in Fig. 3(b). The effect of the spatial filter does not change the spatial specificity of the response but does reduce the amplitude of the OxyHb signal.

Figure 3(d) shows the event triggered averages from the eight individual short channels. The location of the individual short channels is shown in Fig. 2(a). A PC decomposition was performed on the eight individual channels. Subsequent removal of non-neural components utilized either the first PC or the combination of the first two PCs. Figures 3(e) and 3(f) show the effect of removing the first PC and the combined first and second PCs, respectively. The additional effect of removing the second PC was minimal but did not disrupt or reduce the localized response in the occipital lobe. Figure 3(g) shows channels that were determined to be significant for both Oxy- and DeOxyHb in the raw data prior to any filtering. Removing the spatial PC information and the temporal PC information from the short channels produced nearly identical results and are shown in Fig. 3(h). The same channels that were shown to be active using a t-value threshold of 5 prior to applying these two filtering techniques remained significant with the same threshold.

#### 3.1.2 Finger-thumb tapping task

**Fig. 4:**
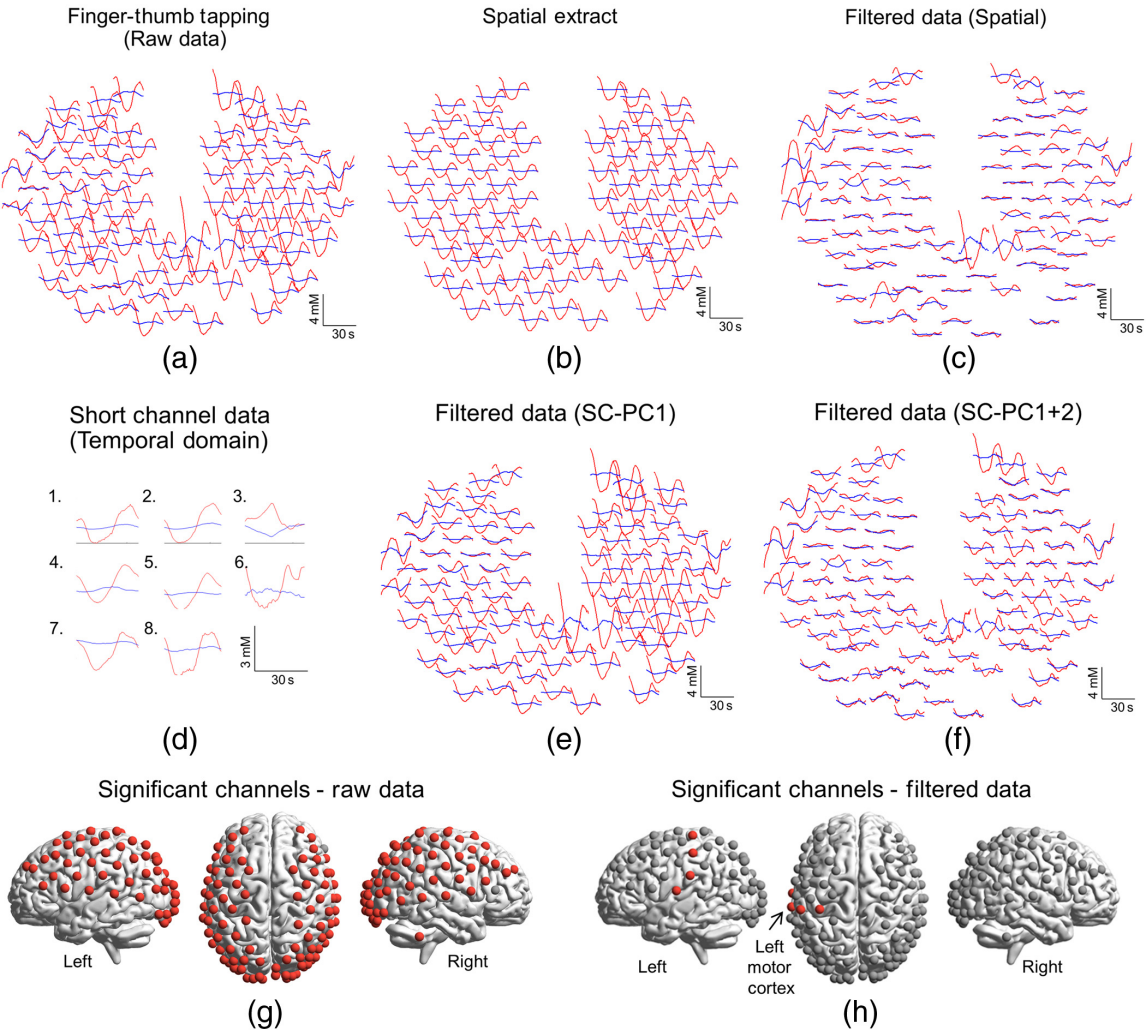
Selected sensory-motor responses from representative participant comparing event-triggered average data between wide distribution of short channels to spatial PC filter. (a) Raw Oxy- and DeOxyHb (red and blue, respectively) signals event-triggered and averaged across the six blocks of the finger-thumb tapping task. The top of the figure represents the front of the head and the bottom of the figure represents the back of the head or the occipital lobe. The expected Oxy- and DeOxyHb hemodynamic separation is not clearly seen in any specific location. (b) The spatial PC filter extracted component. The spatial PC filter determines a global or nonspatially specific component present in the fNIRS signals and removes it based on spatial domain information only. The spatial extract from this representative subject shows a high level of uniformity across the entire cortex for OxyHb signals. (c) The result of removing the extracted non-neural response shown in (b) from (a). The globally uniform nature of the signal is reduced and expected hemodynamic responses are localized to the left sensory and motor cortex. (d) Event-triggered average responses from the eight individual short channels. (e) Result of regressing the first PC of the combined short channels shown in (d) from (a). (f) Result of regressing the first and second PCs of the combined short channels shown in (d) from (a). Channels showing significant response for (g) raw and (h) adjusted data. No differences in the number or spatial location of active channels were found using either spatial or temporal filtering. The results in (h), therefore, represent the results of both filtering techniques as they are equivalent. To determine significance of response single, subject data were modeled using a hemodynamic response function provided in SPM8. In the case that Oxy- and DeOxyHb signals both reach a t-value threshold of 5 or greater, channels are marked as significant (red). Raw data in (g) show responses in all channels with no spatial sensitivity. (h) Both spatial and temporal filtering techniques provide identical results with respect to the number and location of active channels that reach the t-value threshold of 5 for the finger-thumb tapping task for this subject. The data from subject 2 shown in Fig. 4 represent an individual that showed a consistent non-neural response during the active motor task, while other subjects showed similar results, the false positive rate in this subject gained the most benefit from both filtering techniques.

The results of the non-neural component removal comparisons are detailed for the active finger-thumb tapping task in Fig. 4. The same layout and presentation are followed as in Fig. 3. Localized sensory-motor responses are expected for right-handed finger thumb tapping in the left sensory and motor cortices. Localized hemodynamic responses from this subject are not clear from raw data presented in Fig. 4(a). DeOxyHb responses do localize to the contralateral motor area, but OxyHb responses are uniform and present in a similar pattern throughout the superficial cortex. Figure 4(b) shows the Oxy- and DeOxyHb signals extracted from superficial channels using the spatial PC method. Figure 4(c) shows the effect of removing these spatially defined components from the raw data. Event-triggered averaged recordings from the eight short channels for both Oxy- and DeOxyHb signals are shown in Fig. 4(d). Unlike the visual responses shown in Fig. 3(d), the recordings in Fig. 4(d) all appear similar and denote a global or nonlocalized response detected by the superficial channels. Figures 4(e) and 4(f) show similar results using both PCs in the temporal domain, but are less similar compared to the visual results from this subject shown in Figs. 3(e) and 3(f). Only the short-channel PC1 + PC2 extracted result shows localized Oxy- and DeOxyHb separation in the sensory cortex. Figure 4(g) shows all channels having significant activity with respect to the raw data regardless of location. Both temporal and spatial filtering methods produce specificity of localized signals that are significant in the left motor cortex as shown in Fig. 4(h). The results from this exemplar subject were chosen to highlight the extent of the contaminating effects of systemic responses in the raw (i.e., unadjusted) data, especially for OxyHb signals.

### 3.2 Direct Comparison of Superficial Component Extraction Methods for Oxy- and DeOxyHb Signals

**Fig. 5:**
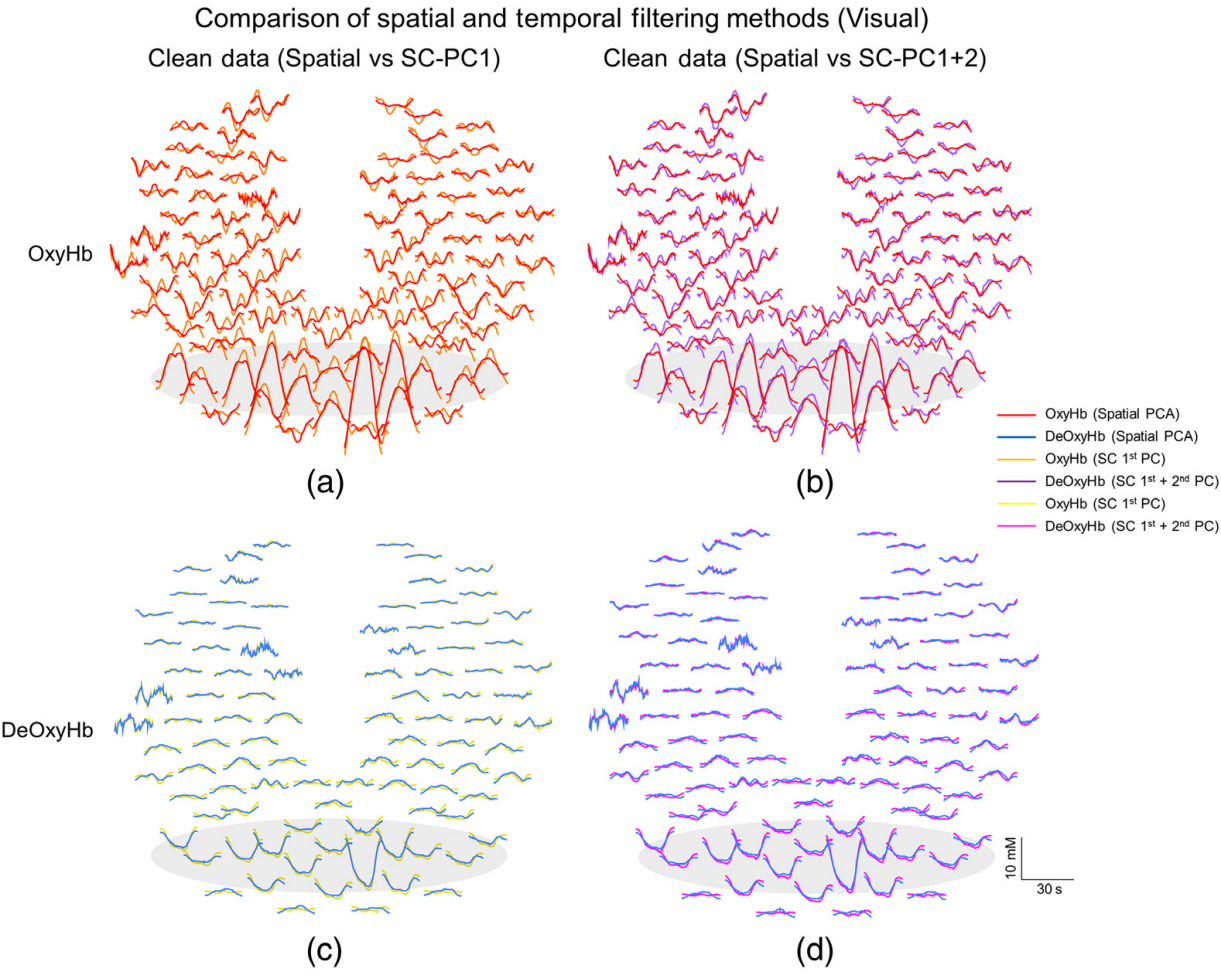
Direct comparison of Oxy- and DeOxyHb responses between spatial and temporal regression methods for visual stimulus from representative participant. (a) Plot of OxyHb responses comparing the resultant adjusted responses from spatial PC regression to short-channel regression using only first PC of combined short channels. The solid red traces represent the spatial PC regression where the orange traces represent the short first PC short-channel regression in the temporal domain. The largest amplitude responses are seen in the back of the head within the occipital lobe. The gray shaded area represents location of significant channels shown in Fig. 2. (b) Plot of OxyHb responses comparing the resultant adjusted responses from spatial PC regression (red traces) to short-channel regression using the combined first and second PCs (purple traces) of combined short channels. Similar adjusted results are shown using both methods and the results also have similar spatial and temporal responses to the comparison seen in (a) with the largest amplitude responses seen in the back of the head within the occipital lobe. (c), (d) Plots of DeOxyHb responses comparing the resultant adjusted responses from spatial PC regression (blue traces) to short-channel regression using only first (yellow traces) or combined first and second PCs (pink traces) of combined short channels. Similar adjusted results are shown using both spatial and temporal regression methods as well as for both PCs of the short channels. The largest amplitude responses are seen in the back of the head within the occipital lobe and correspond OxyHb in location to responses for OxyHb shown in (a) and (b).

Figure 5 compares the results of the two extraction methods for the visual task, using Oxy- and DeOxyHb signals: the spatial filter is plotted together with the temporal filter after short channel first and second PCs have been removed. Figures 5(a) and 5(b) compare spatial results with red traces for OxyHb compared to orange for the short-channel PC1 results and purple for the combined PC1 + PC2 results in the temporal domain. DeOxyHb results are compared in Figs. 5(c) and 5(d). Figures 5(c) and 5(d) compare spatial PC regressed results, with blue traces for DeOxyHb results compared to yellow for the short-channel PC1 results and pink for combined PC1 + PC2 results. The spatial PC filter result is duplicated in both figures for ease of comparison with both short-channel results. Adjusted data with only the first short-channel PC are shown on the left and results with both first and second PCs are shown on the right. The gray shaded area represents the location of the channels found to be significant in Fig. 3. Some variability is seen between the methods for OxyHb signals, but DeOxyHb signals show little difference when comparing adjusted results, regardless of the non-neural extraction method utilized.

**Fig. 6:**
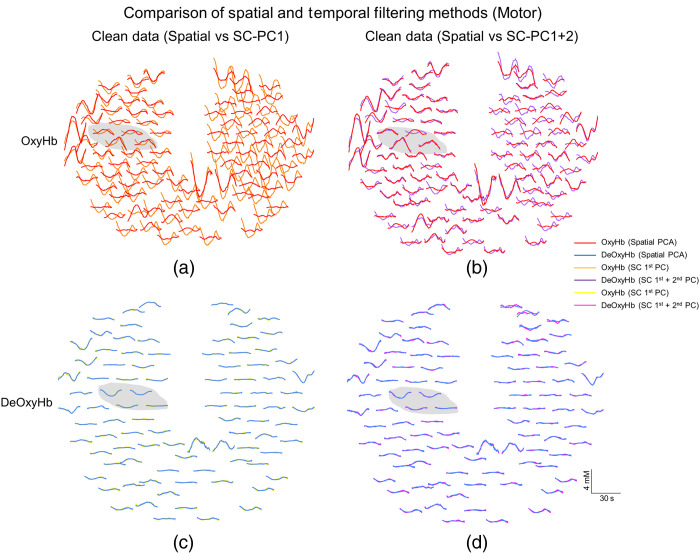
Direct comparison of Oxy- and DeOxyHb responses between spatial and temporal regression methods for finger-thumb tapping from representative participant. (a) Plot of OxyHb responses comparing the resultant filtered responses from spatial PC regression to short-channel regression using only first PC of combined short channels. The solid red traces represent the spatial PC regression where the orange traces represent the short first PC short-channel regression in the temporal domain. The largest amplitude responses are seen in the left sensory and motor cortex using the spatial PC method, but these channels do not show a positive response with the temporal regression of the first PC from the short channels. The gray shaded area represents location of significant channels shown in Fig. 4(h). (b) Plot of OxyHb responses comparing the resultant filtered responses from spatial PC regression (solid red traces) to short-channel regression using the combined first and second PCs (purple traces) of combined short channels. The filtered results from both methods are more similar between the two methods comparing (a) and (b). (c), (d) Plots of DeOxyHb responses comparing the resultant filtered responses from spatial PC regression (solid blue traces) to short-channel regression using only first (yellow traces) or combined first and second PCs (pink traces) of combined short channels. Similar adjusted results are shown using both spatial and temporal regression methods as well as for both PCs of the short channels. Localized responses are present in the gray shaded area.

Figure 6 compares the results of the two extraction methods by directly plotting them for the finger-thumb tapping task. Figure 6 shows Oxy- and DeOxyHb results for the spatial PC filter plotted together with the resultant temporal domain short channel first and second PC removed via short-channel signals. Figures 6(a) and 6(b) compare spatial PC results with red traces for OxyHb results compared to orange for the short-channel PC1 results and purple for combined PC1 + PC2 results. DeOxyHb results are compared in Figs. 6(c) and 6(d). Figures 6(c) and 6(d) compare spatial PC results with blue traces for DeOxyHb results, yellow for the short-channel PC1 results, and pink for combined PC1 + PC2 results. The result using the spatial PC is presented in both figures for each channel for comparison. Adjusted data with only the first short-channel temporal domain PC are shown on the left and results with both first and second PCs are shown on the right. The gray shaded area represents the location of the channels found to be significant in Fig. 4. Similar to results from the visual stimulus task, the DeOxyHb signals show little difference comparing adjusted results, regardless of the extraction method utilized. However, the difference in OxyHb comparing the red traces to the orange and purple show how adding the second PC of the short channels improves the spatial specificity of the response as well as producing a similar result to that of the spatial PC filtering method.

### 3.3 Comparison of First and Second Principal components of Short Channels with Spatial Regression for Whole Head versus Forehead

The channels showing the largest t-value from the GLM analysis within the motor and visual ROI were compared for similarity in signal after performing either spatial or temporal regression. There were no differences in the channel showing the largest t-value between techniques for the seven subjects investigated. A similarity index for each measure comparing how similar the temporal and spatial regressions were are shown in Tables 1 and 2; this metric ranged from 0 to 1 in which a value of 1 represented identical signals and 0 represented no similarity between signals. Oxy- and DeOxyHb results for the widely distributed set of short channels for all participants are compared in Table 1 to the spatially filtered data. The similarity between the spatial PC filter and the first PC of the combined short-channel signals for OxyHb and combined first and second PCs in the temporal domain are shown for both visual and motor tasks. A paired t-test was performed between temporal and spatial regression methods for the channel of best-fit within the ROI for the group results and no significant difference was found (p>0.05). Subject 2 (raw data shown in Figs. 4 and 6) shows a dramatic increase in similarity between temporal and spatial regression techniques when adding PC2 as well as PC1 from combined short-channel recordings for comparing to the spatial regression. The data shown in Figs. 3 and 5 are from subject 3 in Table 1.

**Table 2:** Similarity in results comparing spatial to temporal filtering methods when short channels placed only on the frontal lobe. For all subjects, we compared the similarity of the short-channel regression (PC1 versus PC1 + PC2) to the spatial PC regression method when short channels were placed only on the frontal lobe. The index ranges from 0 to 1 in which a value of 1 represented identical signals and 0 represented no similarity between signals. Oxy- and DeOxyHb comparisons are shown for both visual stimuli and motor tasks.

Forehead only									
Passive-viewing task					Finger-thumb taping task				
Subject	PC1	PC1	PC1 and PC2	PC1 and PC2	Subject	PC1	PC1	PC1 and PC2	PC1 and PC2
	Oxy	DeOxy	Oxy	DeOxy		Oxy	DeOxy	Oxy	DeOxy
1	0.911	0.938	0.955	0.844	1	0.954	0.956	0.997	0.992
2	0.892	0.926	0.984	0.987	2	0.973	0.971	0.981	0.986
3	0.919	0.974	0.965	0.941	3	0.984	0.988	0.996	0.996
4	0.967	0.956	0.982	0.984	4	0.877	0.959	0.987	0.988
5	0.973	0.967	0.996	0.994	5	0.791	0.813	0.943	0.939
6	0.946	0.947	0.962	0.962	6	0.981	0.982	0.983	0.982
7	0.868	0.890	0.989	0.989	7	0.988	0.988	0.987	0.988

Table 2 shows the same responses comparing the task-based spatial filter and the temporal filter using short-channel regression from the second experiment in which the short channels were all placed on the forehead. Paired t-tests comparing subjects and techniques for the group found no significant differences between regression techniques for the channel of best-fit within the ROI. While no significant difference was found between spatial and temporal regression methods for the channel of best-fit within the ROI, short-channel regression using channels on the forehead only did reveal a trend in which adding the second PC from the combined short channels tended to increase the similarity to the spatial regression.

## 4 Discussion

This study compared two methods to separate non-neural components from fNIRS signals employing temporal domain versus spatial domain PC filtering. We compared these filtering methods on data from two fiducial tasks, right-handed finger-thumb tapping and passive viewing of a reversing checkerboard stimulus. These two fiducial tasks have been used previously in multiple neuroimaging modalities to show specificity of location with respect to function in the brain.^21^^,^^53^^,^^72^^–^^74^ The results indicate that a task-based spatial PC filter^28^^,^^53^ compares favorably and performs without significant difference in expected functional and spatial results to short-channel regression, another commonly investigated and cited^39^^,^^41^^,^^43^^–^^46^^,^^51^^,^^52^ temporal domain systemic component removal method. In this study, we compared the results of the spatial filter to regression of the first and second principal temporal components of combined short channels from multiple arrangements on the superficial cortex. In the first experiment, we compared task-based spatial regression to temporal regression of short-channel signals that were evenly placed throughout the whole head, and in the second experiment, we limited short-channel placement to only the frontal lobe and focused their placement on the forehead to assure the highest quality signals from the scalp and other superficial structures. At the group level, we found no significant differences in regressed non-neural components and filtered neural signals in the expected ROI per task. Visual tasks were found to show specific results in the occipital lobe while right-handed finger-thumb tapping produced specific hemodynamic results for both Oxy- and DeOxyHb signals in the contralateral sensory and motor cortices for all subjects as expected (Figs. 3 and 4). Importantly, we found that, for OxyHb signals, there were specific benefits to adding the second PC of the combined short-channel signals for some subjects and no negative effects were seen by regressing the combined signal. DeOxyHb signals showed less contamination from non-neural components and greater similarity between raw and filtered (both spatial and temporal domain) signals than OxyHb signals.

It has previously been shown that short-channel regression can be effectively applied by linear regression of the first PC from a minimum of four short channels placed within the expected ROI.^44^ A recent publication utilized this method as well as adding in a second PC to regress motion artifact.^42^ Their results were less robust for movement artifact regression than systemic components alone, but some benefits were noted. Importantly, in the present study, no negative consequences were shown by additional regression of the second PC of the short channels regardless of the placement of the optodes. We show in the present study that for some subjects with specific types of broad systemic artifact, the first PC may not be sufficient to remove these artifacts. Figures 4 and 6 show the results of one subject where regression of the first PC of short channels is not sufficient to remove systemic components. Only after removal of the combined first and second PCs is the fiducial response evident in the results from this subject. The spatial PC filter performs better than the first PC alone, but upon removal of the combined components, the similarity between the two methods increases as shown in Figs. 4(g) and 4(h) as well as Fig. 6.

It is important to note that Oxy and DeOxyHb signals elicited by the two fiducial tasks revealed responses that were spatially localized as expected. Viewing a reversing checkerboard stimulus produced localized hemodynamic responses in the visual cortex of the occipital lobe, and finger-thumb tapping produced localized responses in the contralateral motor cortex. However, the more active task (compared to passively watching a visual stimulus) often produces additional systemic responses as seen by subject 2 in Figs. 4 and 6. These more pronounced systemic responses are also larger for OxyHb signals compared to DeOxyHb signals, as has been shown previously.^22^^,^^26^^,^^42^^,^^44^^,^^51^ Superficial responses in the passive task are smaller and more variable than they are in the active task. In the active task, this regular task-driven superficial response in the scalp produces a global signal that can be interpreted as a false positive if not filtered or regressed properly. The more random and comparatively small responses in the scalp from the passive task do not model and do not contribute to false positives at the same rate as the active task. Short-channel recordings are obtained during the task responses and the regression of PCs is specific to each task, even when the responses are as variable as those seen in Figs. 3 and 4. The spatial filter we have developed also takes advantage of differential scalp responses to multiple tasks and regresses the spatial component based on task-elicited responses rather than a separate resting state or baseline measure.

The main finding of interest, however, was that there can be large systemic (i.e., contaminating) components in the OxyHb signal (see Fig. 4) and that these non-neural components can be removed successfully using either spatial or temporal filtering, thereby rendering adjusted functional OxyHb signals that are similar in spatial specificity to DeOxyHb signals. Interestingly, even in the subject (Figs. 3 and 6) who had large OxyHb systemic components, the DeOxyHb signals show little systemic effect and neither short-channel temporal regression nor spatial filtering has a large effect on the raw DeOxyHb signals. This is in agreement with previous findings that also show reduced artifact in the DeOxyHb signals compared to OxyHb signals^42^^,^^44^ and may reflect the similarity in origin of DeOxyHb signals to that of BOLD signals acquired using fMRI.^75^ Isolation and removal of specific contributions and sources that generate additional components in the OxyHb signals remain a challenge, but both temporal and spatial domain methods described here can be utilized to remove similar non-neural signals. Another challenge is the variability between subjects with respect to scalp hemodynamics. Variability in hemodynamics can happen day to day and even based on season.^76^ Because of this, it is difficult to predict or repeat specific scalp responses within or across subjects. In this study, not all subjects displayed a large scalp response in active or passive tasks. However, we show here that for subjects that did display large scalp derived hemodynamic responses, both temporal and spatial regression strategies are sufficient to filter these responses from neural signals in long channels.

The results of this study also indicate very close similarities in the components removed by short channel and spatial regression strategies (Figs. 5 and 6). While short-channel regression is in the temporal domain and the spatial method uses spatial domain, it is possible that both are indeed regressing similar information from task-evoked sympathetic arterial responses in the scalp and superficial tissue. One goal of our spatial filter was to perform the regression in the spatial domain instead of the temporal domain in the case that systemic components were completely nonorthogonal to the task. In this case, restricting the regression to spatially diverse regions argue that the response is global and non-neural. Temporal regression of nonorthogonal responses may reduce real responses, but in this study, we did not find evidence of this. The similar results produced by the different techniques do support that they are regressing similar information from long-channel data regardless of spatial or temporal domain. The shared spatial information may largely represent superficial hemodynamics in the same way that path length of 8-mm short channels is designed to target responses from the scalp. We argue that this is likely the case and that both regression methods are targeting non-neural and broad responses that do not localize. The multiple component short channel and spatial regressions we show here are both able to remove non-neural superficial responses and produce fiducial responses that are in accordance with known regions for finger tapping and visual stimuli; however, we cannot claim that we are regressing all systemic components from long channels. A recent study has shown that scalp hemodynamics have additional heterogeneous components in addition to the global homogeneous behavior.^50^ The study showed similar results as we have found in the present report supporting multiple scalp regressors, but it was also suggested that short-channel regression can be more effective when additional measures of physiology, such as Mayer waves, are included in the regression.

A secondary finding of interest in this study is whether the placement of short channels can improve temporal signal regression results. We asked whether placement on the frontal lobe versus broadly arranged on the whole head as in previous studies^24^ would result in superior extraction of non-neural components in the OxyHb signal. This setup is considerably easier than whole head placement of short channels and can decrease set-up time significantly. The results of this part of the experiment support the previous findings of Sato et al.^44^ and suggest that combined short-channel information may be sufficient to remove superficial hemodynamics when localized only on the frontal regions not covered by hair. We agree with Sato et al. that a minimum of four channels should be utilized to obtain at least one PC but, in addition, we argue that at least four channels are necessary to determine the second PC and the combined first and second PC regression does not harm the data and likely assures additional components are regressed from all subjects as has been suggested.^50^ The NIRx NIRScout system had a total of eight short channels that were placed inside of the elastic cap. It was more difficult to assure every short channel made good scalp contact in the whole head experiment, especially with subjects that had thick dark hair. If channels did not make good scalp contact, they tended to show high-frequency noise, which did not contribute to the PCs utilized in the regression. In the forehead placement of channels, it was faster and easier to assure that short channels were making scalp contact even though regression was not as efficient as whole head placement.

## 5 Conclusions

In summary, we have shown that these spatial and temporal signal processing methods produce similar results in the context of the two fiducial tasks utilized in this study and do not produce significant differences in the resultant filtered signals. The assumption that signals recorded from short channels contain only limited information from the anatomical space superficial to the cortical gray matter is supported by the results presented here. The spatial PC filter does not utilize temporal information, rather only spatial similarities in the signals across multiple contiguous channels for regression. The similarities in the results with spatial and temporal filtering support the conclusion that both techniques are removing similar components from the 3-cm channels. Both of these methods can be utilized to compensate for false negatives as well as false positives (Fig. 4) in fNIRS experiments. While neither technique can be considered to perfectly remove all non-neural components from the hemodynamic signal, we have shown here that both techniques remove similar components and that spatially specific fiducial results are also found with both techniques. This is an important finding because not all functional fNIRS devices have short-channel hardware as an option, but the spatial PC filter can effectively be utilized on optode arrays as small as 3×3.^53^ A future direction of this research aims to further examine emotional and cognitive influences on scalp signals as well as responses to simple passive versus active fiducial tasks in a larger group to determine how these regression techniques affect data quality at the group level.

Based on these findings, we conclude that fNIRS signals, and especially OxyHb signals, are optimally processed by some method of superficial hemodynamic removal technique regardless of whether it utilizes temporal or spatial domain information. We show that both spatial PC filtering as well as short-channel regression in the temporal domain can be effectively utilized to remove superficial non-neural hemodynamics from fNIRS signals.

## Code, Data, and Materials Availability

The data and MATLAB code from this article will be made available upon request at fmri.org by contacting the corresponding author.
